# *Bifidobacterium animalis* subsp. *lactis* BB-12 Improves the State Anxiety and Sports Performance of Young Divers Under Stress Situations: A Single-Arm, Prospective Proof-of-Concept Study

**DOI:** 10.3389/fpsyg.2020.570298

**Published:** 2021-01-13

**Authors:** Weizhong Dong, Ying Wang, Shuaixiong Liao, Wei Tang, Li Peng, Gang Song

**Affiliations:** ^1^Research Centre for Exercise Detoxification, College of Physical Education, Southwest University, Chongqing, China; ^2^Key Lab of Physical Fitness Evaluation and Motor Function Monitoring, College of Physical Education, Southwest University, Chongqing, China

**Keywords:** probiotics, state anxiety, gut microbiota, sports performance, diver

## Abstract

**Background:**

Athletes will increase their state anxiety under stress situations, which will lead to the decline of sports performance. The improvement of anxiety by probiotics has been reported, but there is a lack of research in the athlete population. The purpose of the current study is to explore the effectiveness of probiotics in improving athletes’ state anxiety and sports performance under stress situations.

**Methods:**

We conducted this single-arm study in Chongqing Institute of Sports Technology. In the 8-week study, 21 Chongqing young divers (mean age: 9.10 ± 1.80) were given probiotic *Bifidobacterium animalis* subsp. *lactis BB-12* (1 × 10^9^ colony-forming units/100 g) daily. The state anxiety and sports performance of athletes were measured before, during, and after the intervention, and the gut microbiota of athletes was measured before and after the intervention.

**Results:**

The intervention results showed that cognitive state anxiety, somatic state anxiety, and anxiety emotion were improved (cognitive: *Z* = −3.964, *P* < 0.001; somatic: *Z* = −3.079, *P* = 0.003; anxiety: *Z* = −2.973, *P* < 0.001). In terms of gut microbiota, the intervention did not change the gut microbial composition (such as α diversity and β diversity) but increased the abundance of *Bifidobacteriaceae*. At the 8th week, the performance of athletes under stress was significantly improved (χ^2^ = 7.88, *P* = 0.019).

**Limitations:**

First of all, due to the restriction of the number of subjects in this study, there was no control group. Secondly, although the athletes’ diet was recorded in this study, the influence of this factor on gut microbiota was not eliminated. Finally, the anxiety level of the athletes in this study was obtained through a self-report, lacking physiological data in state anxiety.

**Conclusion:**

The results show that probiotics intervention can improve the state anxiety of athletes under stress situation and improve the performance of athletes under stress situation.

## Introduction

The intake of probiotics has some beneficial effects on individuals. In addition to the potential applications of probiotics in anti-tumor, anti-diabetic, anti-obesity, anti-inflammatory, anti-cancer, antiallergic, and angiogenic activities and other aspects mentioned in previous studies, it was also found that probiotics may affect the brain and the central nervous system (CNS) (George Kerry et al., 2018). The “microbiota–gut–brain axis” is an interactive, bidirectional communication established by the exchange of regulatory signals between the gastrointestinal tract and CNS (Mayer et al., 2015). Early human studies suggest that altering the microbiota with beneficial bacteria, or probiotics, can lead to changes in brain function as well as subjective reports of mood (Tillisch, 2014; Mayer et al., 2015). Considering the applicability of probiotics, several studies have focused on the benefits of probiotics for athletes. Probiotics have been shown to play a role in improving the athletes’ immune xsystem, oxidative stress, metabolism, and cognitive ability (Jäger et al., 2019). After the intake of *Bifidobacteria*, the maximum oxygen uptake of swimmers was increased (Salarkia et al., 2013), and the intake of *Bifidobacteria* in marathon athletes could reduce their gastrointestinal symptoms and plasma endotoxin levels after the competition (Roberts et al., 2016; Pugh et al., 2019). In addition, studies have found that *Bifidobacteria* plays a positive role in improving muscle performance (Ibrahim et al., 2018) and maintaining muscle tone (Jäger et al., 2016a). In addition, the intake of probiotics, such as *Bifidobacteria*, can stimulate the proliferation of beneficial metabolites (such as short-chain fatty acids) of specific microorganisms, thus improving the metabolism, immunity, and barrier function of athletes (Pane et al., 2018).

Athletes’ cognition may affect their performance. It has been reported that state anxiety has an impact on the performance of technical sports (Gao and Yan, 2010). State anxiety refers to a kind of anxiety that is short-lived and easily affected by the environment (Walkenhorst and Crowe, 2009). This is due to the pressure brought by the actual situation. Intense competition as well as other factors, such as the mutual restriction relationship between competitors and the immediate awareness of results in sports competitions, has made the anxiety experience very common for athletes during training and competition (Strahler et al., 2010). Previous studies have shown that there is a significant negative correlation between state anxiety and the performance of athletes in technical events (De Pero et al., 2016; Mehrsafar et al., 2019), and an increase in state anxiety will lead to a decline in performance (Walter et al., 2019). It was found that anxiety can inhibit the athletes’ automatic behavior (Eysenck et al., 2007).

Furthermore, the study on *Bifidobacteria* found that *Bifidobacteria* has a positive effect on the anxiety state of different populations. Taking probiotics composed of *Lactobacillus helveticus* R0052 and *Bifidobacterium longum* R0175 can significantly reduce the anxiety level of healthy volunteers (Messaoudi et al., 2011); the DASS-42 score of adult healthy women is significantly negatively correlated with *Bifidobacteria* (Taylor et al., 2019), and the elderly can improve the mental state of anxiety and depression by supplementing *Bifidobacteria* and moderate resistance training (Inoue et al., 2018). In addition, taking *Bifidobacteria* can improve anxiety in patients with irritable bowel syndrome or multiple sclerosis (Ma et al., 2019; Salami et al., 2019).

The benefits of probiotics in athletes have been reported, but there are few positive results in terms of cognition and emotion. Sawada et al. found that CP2305 supplementation could improve the anxiety and depression scores of long-distance runners (Sawada et al., 2019). Unfortunately, this study did not discuss changes in exercise performance after probiotics intake. In another study, probiotic intervention successfully reduced the pressure perception of athletes and tested the sports performance of mobilization, but no difference was found (Salarkia et al., 2013). Whether probiotics can alleviate the anxiety of athletes, especially the state anxiety under stress, or the change will affect the performance of sports is what this study aimed to explore. In this study, we selected athletes of technical events (diving) that are greatly affected by state anxiety. According to previous research, state anxiety is related to perceived pressure (Hsu et al., 2019). Therefore, this study conducted 8 weeks of intervention for diving athletes. We designed the stress situation and observed the changes in state anxiety and sports performance of athletes taking *B. animalis* subsp. *lactis* under stress.

## Materials and Methods

### Subjects and Procedure

In this study, 21 divers from the Chongqing Sports Technical School were selected. All of them were male and ate and lived together because of the training. The exclusion criteria were as follows: (1) recent use of antibiotics, (2) diarrhea and insomnia in the week before the experiment, (3) habit of taking probiotic supplements, and (4) inability to eat and live with other athletes for personal reasons during the experiment. It should be noted that, due to their age, these athletes have not yet received training on how to relieve pre-competition anxiety.

This study is a single-arm study, which requires athletes to conduct sports performance tests under pressure before, during, and after the intervention, complete the measurement of state anxiety and anxiety before the test, and obtain the athletic performance of athletes. We also measure the gut microbiota of athletes before and after the intervention to compare the changes of gut microbiota. The recipes of the athletes are circulated weekly. We have recorded the recipes of the athletes for a week and submitted them to the Chinese clinical trial center with the code chictr1900024119.

During the experiment, the athletes were preparing for the National Children’s Diving Championships in August. This study was approved by the ethics committee of the Southwest University Hospital in Chongqing, China, with the code 201903, and recorded in the Chinese clinical trial center with the code ChiCTR1900024119.

### Supplementary Beverages

The yogurt used in the study (Mengniu Dairy (Group) Co., Ltd.) was rich in *Bifidobacterium animalis* subsp. *lactis* BB-12 (1 × 10^9^ colony-forming units (cfu)/100 g), *Lactobacillus bulgaricus* (1 × 10^8^ cfu/100 g), and *Streptococcus thermophilus* (1 × 10^8^ cfu/100 g) according to the Chinese National Food and Drug Administration domestic health food approval certificate (no.: 2015B0306). Considering the age of the participants, the daily intake was recorded by the experimental personnel to ensure the intake compliance rate.

### Measurement of Emotion and State Anxiety

The participants’ emotions were measured using the Piers–Harris Children’s Self-Concept Scale (PHCSS). The scale was developed by American psychologists Piers Harris and DBEV in 1969 and revised in 1974. This scale is mainly used to evaluate children’s self-awareness. There are 80 items in total, which can be divided into six subscales: behavior, intellectual and school status, physical appearance and attributes, anxiety, popularity, and self-contentment (Alexopoulos and Foudoulaki, 2002). In the study of different types of adolescents in China, the anxiety subscale of the scale shows good discrimination, reliability, and validity (Li et al., 2010; Huang et al., 2016; Zhao et al., 2016). In this study, the anxiety subscale was selected, with 14 items in total. In this study, the test–retest reliability of the PHCSS was 0.804.

The state anxiety of the competition situation was measured with the Mental Readiness Form-3 (MRF-3). MRF-3 is composed of three parts, and each part is a 100-mm straight line. The two ends of the body anxiety line represent “worried–not worried,” while those of the cognitive anxiety line represent “tense–not tense” and those of the state self-confidence line represent “confident–unconfident.” According to the actual situation, athletes marked their own suitable situations on the line. This scale had a high homogeneity with the commonly used Competition State Anxiety Inventory-2 (CSAI-2), among which the consistency coefficients with CSAI-2’s body anxiety scale and cognitive anxiety scale were 0.69 and 0.76, respectively (Mesagno et al., 2019). In this study, only the physical and cognitive state anxieties of athletes were measured.

The athletes are asked to attend a meeting before measuring their performance in a simulated real game (see the details in “Section 2.5”). The purpose is to further arouse the athletes’ pre-competition anxiety. The athletes were asked to fill in the questionnaire immediately after the meeting. All questionnaires were completed in the Diving Hall of Chongqing Sports Technical School.

### Analysis of Gut Microbiota

#### Sample Collection and DNA Extraction

Fecal samples were collected at the Affiliated Hospital of Southwest University and frozen at −80°C within 3 h of sampling. DNA extraction was performed using a QIAamp Fast DNA Stool Mini Kit (Qiagen, CA, United States). The concentration of bacterial DNA was measured using a NanoDrop 2000 spectrophotometer (Thermo Scientific, Waltham, MA, United States).

#### High-Throughput Sequencing

The bacterial communities in the fecal samples were investigated by Illumina MiSeq high-throughput sequencing. The V3 and V4 regions of the 16S rDNA gene were selected for PCR. The primers were barcoded as 338F (5′-ACTCCTACGGGAGGCAGCAG-3′) and 806R (5′-GGACTACHVGGGTWTCTAAT-3′), where the barcode was an eight-base sequence unique to each sample. The 20-μl PCR reaction mixture was composed of 4 μl of 5 × FastPfu buffer, 2 μl of 2.5 mM dNTPs, 5 μM each of forward and reverse primer, 0.4 μl TransStart Fastpfu DNA Polymerase (TransGen Biotech, Beijing, China), and 10 ng DNA template. The following cycling parameters were used: maintenance at 95°C for 2 min, 27 cycles of 95°C for 30 s, 55°C for 30 s, and 72°C for 30 s, and a final extension at 72°C for 5 min. Triplicate reaction mixtures were pooled for each sample, purified using an AxyPrep DNA gel extraction kit (Axygen, Union City, CA, United States), and quantified using a QuantiFluor-ST fluorescence quantitative system (Promega, Madison, WI, United States). Amplicons from different samples were sent out for sequencing on an Illumina MiSeq platform at Shanghai Majorbio Bio-Pharm Technology Co., Ltd. (Shanghai, China).

#### Processing Sequencing Data

Raw FASTQ files were demultiplexed and quality-filtered using Quantitative Insights Into Microbial Ecology (QIIME) (version 1.9.1) with the three criteria mentioned in Song et al. (2017). Operational taxonomic units were clustered with a 97% similarity cutoff using UPARSE (version 7.12), and chimeric sequences were identified and removed using UCHIME. The taxonomy of each 16S rRNA gene sequence was analyzed using the RDP classifier against the Silva (SSU128) 16S rRNA database using a confidence threshold of 70%. Relevant data from the gut microbiota were uploaded to the National Omics Data Encyclopedia (NODE^1^, Sample ID: OEP001026).

### Simulations of Real Competition

Stress can lead to an increase in state anxiety in athletes. Since the competition field is not open to researchers, it is impossible to measure the anxiety levels of the athletes in a real competition environment. Geukes et al. stated that the use of simulations of real competitions was a better alternative (Geukes et al., 2013). Therefore, this study constructed a scenario to simulate a real competition (stress situation). In this scenario, the pressure setting referred to the research of Wang et al. (2018). We chose the external (referees, spectators) and internal pressures (enhance the athletes’ attention to the game) to wake the state anxiety of athletes. We scheduled the test on the last day of week 0, week 4, and week 8 to ensure that the time between each test was equal. Each test is aimed to evaluate the mental state first and then to evaluate the sports performance. All exercise performance tests under stress were performed at the Diving Hall of Chongqing Sports Technical School.

In addition to simulating the competition situation, we also counted the athletes’ performance during training. Training situations are considered less stressful. This study aimed to eliminate the possible effect of long-term training on the results of this study.

### Sports Performance

To avoid the ceiling effect, after communicating with the coach, four basic movements in the juvenile diving competition were chosen as the basis for measuring the athletes’ performance. In this study, we invited three national referees to score the performance of the diving athletes in the simulation competition. The scoring standard was consistent with that of an official competition (the full score was 10, and the average score of the three referees was the final score). The athlete will know the referee’s score the first time after finishing the movement.

### Data Analysis

We did not assume that our relatively small sample had a normal distribution and used the values of our samples without transformation for the purpose of enabling a clear interpretation. We used the Wilcoxon rank sum test to analyze the scale scores and sports performance at different times. Sobs, Shannon, Simpson, Ace, Chao index, and principal coordinate analysis (PCoA) were performed using QIIME 1.9.1 (Mitter et al., 2017). The Wilcoxon rank sum test was used to measure the differences in gut microbiota between groups. Benjamini and Hochberg’s false discovery rate (FDR) was used to adjust the results, and significant associations were considered below an FDR threshold of 0.05 (CI bootstrap 0.95). Statistical analysis was carried out using GraphPad Prism V8.3.0 (La Jolla, CA, United States), R statistical package (V.3.6.3).

## Results

### Physical Activities of the Participants

The characteristics of the athletes are shown in Table 1. During the experiment, there was no loss of participants, and no reports of adverse events due to probiotic yogurt were reported. The compliance of the participants is reflected by the test drink intake rate in the table. In 8 weeks, the athletes trained every day, and the training plan was recorded by the coaches. Since diving athletes were not allowed to carry wearable equipment to measure the activity during training, the experts were invited to review their training plans to ensure that there was no impact on the results of this experiment due to excessive exercise.

**TABLE 1:** Characteristics of the subjects.

Parameters	Participants
Age (years)	9.10 ± 1.80
Height (cm)	137.34 ± 9.68
Body weight (kg)	30.23 ± 5.04
BMI (kg/m^2^)	15.93 ± 1.35
Exercise years	3.90 ± 1.00
Test drink intake rate (%)	94.30 ± 3.30

*BMI, body mass index; cm, centimeter; kg, kilogram. Values are represented as mean ± SD.*

### Effects of *B. animalis* subsp. *lactis* on Mental State

**FIGURE 1:**
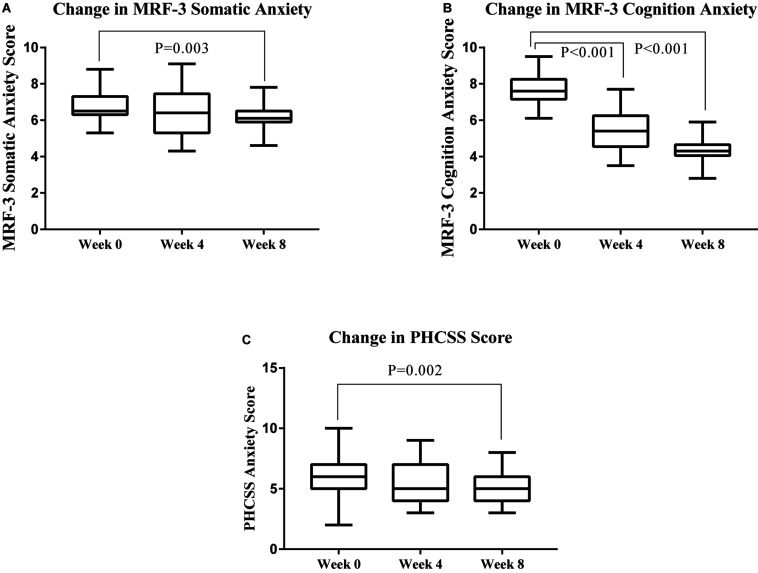
Effects on the changes of mental state during the experimental period as determined by questionnaires; somatic anxiety score **(A)**, cognitive anxiety score **(B)**, and anxiety emotion score **(C)**. Data were analyzed using Wilcoxon matched-pairs signed-ranks test between the groups and the *p*-value is shown in each panel.

The results of the questionnaires are summarized in Table 2. During the experiment, the athletes participating in the experiment were preparing for the National Junior Diving Championships in August. A simulation match was designed on week 0, week 4, and week 8 to collect the state anxiety and emotion of athletes under stressful conditions. MRF-3 was used to evaluate the state anxiety of athletes, and PHCSS was used to evaluate emotions. The changes in the athletes’ mental state are shown in Figure 1. Compared to week 0, the cognitive state anxiety of athletes significantly reduced at week 4 (*Z* = −3.964, *P* < 0.001), and the somatic state anxiety significantly reduced at week 8 (somatic: *P* = 0.003; anxiety: *Z* = −3.079, *Z* = −2.973, *P* = 0.002).

**TABLE 2:** Results of Mental Readiness Form-3 (MFR-3) and Piers–Harris Children’s Self-Concept Scale (PHCSS).

Questionnaire	Week 0	Week 4	Week 8
Somatic (MRF-3)	6.76 ± 0.80	6.44 ± 1.29	6.15 ± 0.80
Cognition (MRF-3)	7.62 ± 0.84	5.38 ± 1.16	4.30 ± 0.71
PHCSS	6.19 ± 1.80	5.57 ± 1.75	5.10 ± 1.37

*Values are represented as mean ± SD.*

### Effects of *B. animalis* subsp. *lactis* on Gut Microbiota

The α diversity index can reflect the richness and diversity of the microbial community. In this study, we analyzed the gut microbiota α diversity of the athletes before and after the intervention, and the results are shown in Table 3. There was no significant difference in several common α diversity indices before and after the intervention.

**TABLE 3:** Test results of α diversity index.

Estimators	Week 0	Week 8	*P-*value	*Q-*value
Sobs	35.33 ± 6.51	37.19 ± 6.06	0.412	0.495
Shannon	1.68 ± 0.36	1.82 ± 0.27	0.268	0.495
Simpson	0.30 ± 0.11	0.24 ± 0.08	0.092	0.495
Ace	44.57 ± 13.09	44.92 ± 10.11	0.725	0.725
Chao	40.45 ± 10.31	42.23 ± 8.25	0.372	0.495

*Several common α diversity index results are listed in the table. Values are represented as mean ± SD. The *Q-*value represents the false discovery rate value.*

**FIGURE 2:**
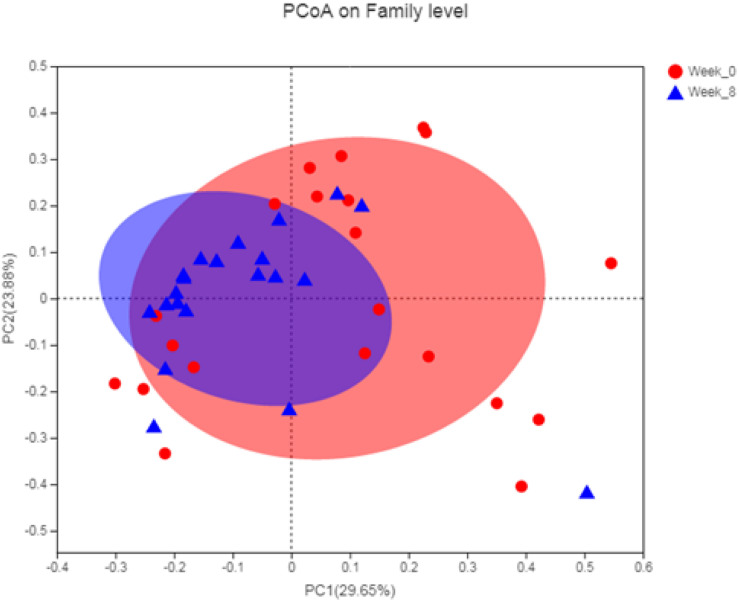
Analysis of β diversity of gut microbiota in athletes before and after intervention.

For the β diversity of the gut microbiota of the athletes before and after the intervention, PCoA was selected for analysis, and the results are shown in Figure 2. Before and after the intervention, there was no significant difference in the composition of the gut microbiota community.

**FIGURE 3:**
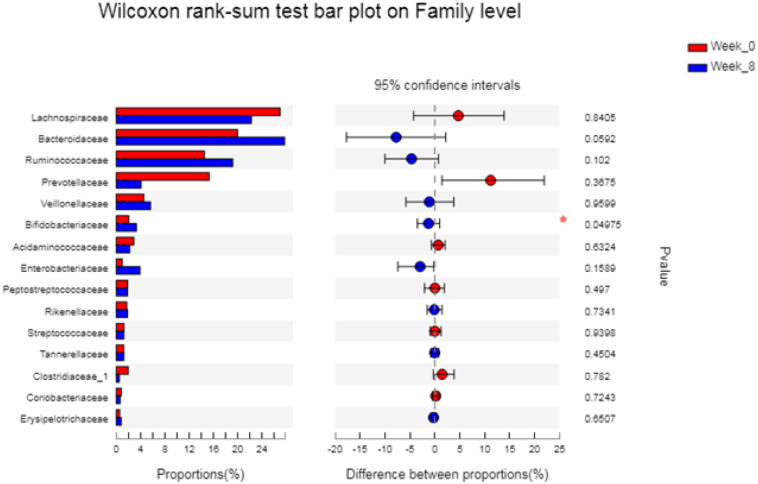
The differences between the groups of gut microbiota. The vertical axis (left) shows the name of the bacteria at the family level. Each column that corresponds to a family represents the average relative abundance of each family in each sample group, and different colors represent the different groups. The middle area represents the difference in abundance percentage between the two groups within the set confidence interval. The colors of the dots represent the groups in which family abundance occupies a large proportion. The I interval on the dot represents the upper and lower limits of the difference. *p < 0.05.

Figure 3 shows the differences before and after the intervention. At the family level, the abundance of *Bifidobacteriaceae* in athletes after the intervention was significantly higher than that before the intervention (*P* = 0.05).

**FIGURE 4:**
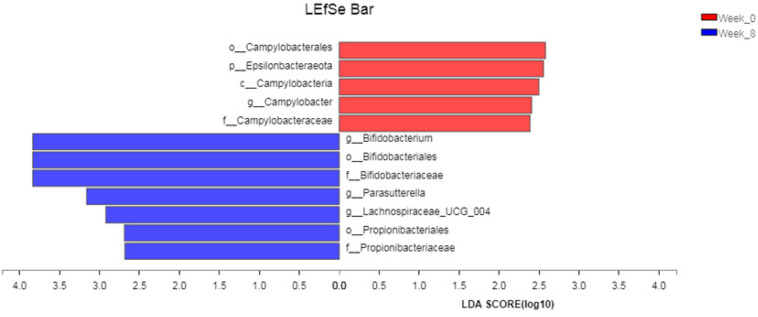
LEfSe Analysis. The LDA threshold is set to 2, and one against all (less strict) is selected for multi group comparison.

Linear discriminant analysis effect size (LEfSe) analysis was used to test the abundance difference of gut microbiota at different levels before and after intervention. It was found that, after 8 weeks of yogurt intake, the abundance of *Parasutterella*, *Lachnospiraceae*, and *Propionibacteriaceae* also changed significantly. The results of the LEfSe analysis are shown in Figure 4.

### Effects of *B. animalis* subsp. *lactis* on Athletes’ Performance

**FIGURE 5:**
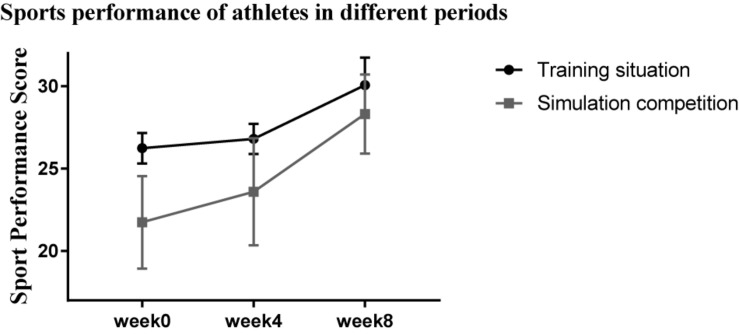
Broken line chart of athletes’ performance under different pressures. week0 Training situation: 26.24 ± 0.93, Simulation competition: 21.74 ± 2.81; week4 Training situation: 26.80 ± 0.92; Simulation competition: 23.59 ± 3.25; week8 Training situation: 30.07 ± 1.67; Simulation competition: 28.32 ± 2.40.

**FIGURE 6:**
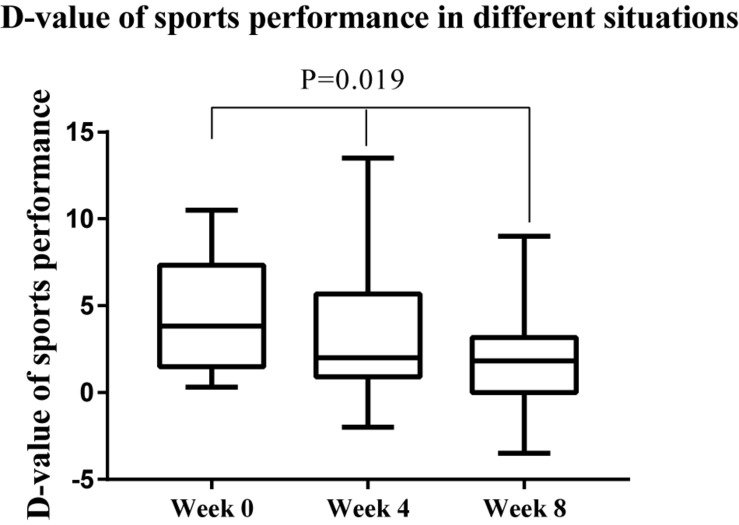
Changes of *D*-value of sports performance in different situations. *D*-value = Training situation score minus Simulations of real competitions score.

The performance of the athletes under stress is shown in Figure 5. At weeks 0, 4, and 8, the performance tests under simulated real-competition environment and training environment were conducted. To show that the enhancement of athletes’ sports performance in simulated competition was not related to the accumulation of training, we also reported the sports performance scores in the training situation. It can be seen in Figure 5 that, at the end of the experiment (week 8), the performance in the simulation competition was closer to that of the training environment. Furthermore, Friedman test was conducted on the difference between the training and simulated competition situations. The results are shown in Figure 6. There were significant differences in sports performance at different time points (χ^2^ = 7.88, *P* = 0.019), indicating that, with the extension of time, the sports performance of athletes in the simulation competition gradually tended to be consistent with the training situation.

## Discussion

Under stress, exercise performance will decrease due to the increase of state anxiety. This phenomenon has been noticed by many researchers. When studying the influence of quiet eye training on the performance of golfers, Samuel et al. found that a stress situation can lead to an increase of state anxiety and a decline of golfers’ putt performance (Vine et al., 2011). Wilson et al. found that, under a pressure situation, football players are more likely to pay attention to the existence of goalkeepers, which leads to an increase of perceived anxiety and a decrease of shooting accuracy (Wilson et al., 2009). It should be pointed out that the anxiety of the athletes decreased at week 8, which may be affected by the habituation effect. Although this kind of simulation competition is not created for the purpose of research alone, that is, when there is no intervention, they will hold simulation competition regularly to observe the recent research results. However, we did not collect the anxiety state of the athletes participating in a simulation competition in other periods, so we cannot exclude the influence of habituation effect.

In this study, we tried to observe the change of athletes’ state anxiety and its effect on sports performance through the intervention of probiotics. The results show that both somatic state anxiety and cognitive state anxiety are reduced from the previous serious anxiety level to the middle and low anxiety level after intervention, and the performance of the athletes under stress situation has been improved. This shows that the improvement of anxiety will have a positive impact on the performance of the athletes. There are many theories to explain the relationship between sports performance and state anxiety. According to the Attention Control Theory, anxiety interferes with the inhibition and refreshing of the central executive system of athletes’ working memory so as to affect the performance of athletes (Mullen et al., 2005).

In real life, due to the need for long-term training, the diet of athletes is often controlled, which leads to a decrease in the abundance of probiotics in the gastrointestinal tract (Jang et al., 2019) and may also lead to a decrease in the α diversity of the gut microbiota, which will increase the risk of the athletes to suffer from gastrointestinal diseases (Clarke et al., 2014). The diversity of the gut microbiota has been shown to be positively correlated with cardiopulmonary fitness (Estaki et al., 2016). Taking yogurt containing probiotics can not only enhance the sense of fullness and avoid overeating but can also improve the probiotic abundance in the body (Zhang et al., 2019). In this study, yogurt containing probiotics was used to intervene in the gut microbiota of athletes. This study explored the effect of *B. animalis* subsp. *lactis* on athletes’ perceived anxiety and performance under stress. Diet is an important factor in this type of research. In this study, the athletes of Chongqing diving team were recruited. They stayed together and ate three meals a day in the canteen of Chongqing Institute of Sports Technology. We have recorded the athletes’ daily recipes, but we still cannot rule out the impact of diet on gut microbiota because we do not record every athlete’s daily diet choice. To avoid the influence of very few samples on the reliability of the experiment, instead of a double-blind, randomized, and placebo-controlled clinical trial, a single-arm study was selected.

After 8 weeks of probiotic supplementation, some changes could be seen in the gut microbiota of the athletes. We found that the supplementation of *B. animalis* subsp. *lactis* did not change the α and β diversity of the gut microbiota in the athletes, but it improved the abundance of *Bifidobacteriaceae* in the athletes. A change in the gut microbiota may affect an individual’s mood. Many studies support that the supplementation of probiotics results in the improvement of anxiety in college students, obese people, and candidates under stress (Hadi et al., 2019; Nishida et al., 2019; Tran et al., 2019). A study in 2018 showed that probiotics could improve the cognitive function of athletes during off-season endurance training (Carbuhn et al., 2018). Luna et al. stated that probiotics could affect the gut microbial community, affecting the pathway of the microbial community to the brain, thus affecting cognition and behavior (Luna and Foster, 2015). When athletes are under pressure, the gastrointestinal tract releases hormones, such as gamma-aminobutyric acid (GABA) and short-chain fatty acids (SCFAs), to participate in the regulation of the hypothalamic–pituitary–adrenal axis (Clark and Mach, 2016). State anxiety is affected by the force of stress. Fond et al. suggested that probiotics could interact with the gut–brain axis to regulate the effect of stress on gut microbiota (Fond et al., 2015). Probiotics are not only related to the production of SCFAs (Pane et al., 2018) but have also been shown to reduce the mRNA expression of the GABA receptor and c-fos in the brain by regulating the gut–brain axis *via* the vagus nerve pathway (Kato-Kataoka et al., 2016). Many studies have shown that probiotics can regulate brain function and stress state (Takada et al., 2016; Molina-Torres et al., 2019).

This study focused on the improvement of sports performance of athletes after taking probiotics. In strength training, the consumption of protein leads to an increase in hydrogen sulfate, which may damage intestinal health (Jäger et al., 2016b). The intake of probiotics can directly improve the health status of athletes and indirectly improve their performance (Leite et al., 2019). In addition, supplementation of probiotics can improve the oxidative stress of triathlon athletes and their sports ability (Michalickova et al., 2018; Huang et al., 2019). However, most of these results are based on the physiological conditions of the athletes, and the conclusion of sports performance is speculated. In this study, probiotics could improve the state anxiety of athletes. With the decrease in state anxiety, the performance of athletes in simulated competition improved. Here we emphasize the influence of anxiety on sports performance. Diving is a kind of technical project that requires a highly technical action (Xu et al., 2010). In such sports, the pressure causes the athletes to focus more on the process of sports, consciously control the execution process of sports, try to improve the efficiency of completing actions, and lead to the execution process that has formed automation to be blocked (Wang, 2004).

In this study, we improved the state anxiety of athletes under stress by regulating the gut microbiota and observed that, with the decline of state anxiety, the performance of simulated competition gradually improved, which enriched the related research on probiotics and sports performance. For technical events, the performance of sports is significantly related to the level of anxiety of the athletes. However, this study was not a randomized controlled trial, and anxiety was measured by subjective self-report. Another drawback is that only microbial diversity analysis was conducted for gut microbiota, not metagenome and metabonomics analysis. In future research, we should design randomized controlled trials, include more volunteers, collect physiological data related to anxiety (such as salivary cortisol), and perform metagenome or metabonomics analysis of the gut microbiota to clarify how probiotics affect athletes’ anxiety under stress.

## Conclusion

In summary, this study showed that the daily intake of *B. animalis* subsp. *lactis* was effective in reducing the state anxiety of athletes and could improve the performance of athletes under stress. In addition, the intake of *B. animalis* subsp. *lactis* increased the abundance of *Bifidobacteriaceae* in athletes. This study demonstrated the potential of probiotics to improve mental state and performance in athletes under stress.

## Data Availability Statement

The original contributions presented in the study are included in the article/supplementary material, further inquiries can be directed to the corresponding author/s.

## Ethics Statement

The studies involving human participants were reviewed and approved by Southwest University Hospital Ethics Review Committee. Written informed consent to participate in this study was provided by the participants’ legal guardian/next of kin.

## Author Contributions

WD, LP, and GS conceptualized and designed the research. WD and YW performed the experiments. WD and SL analyzed the data. WD and GS plotted the figures. WD drafted the manuscript. All the authors interpreted the experimental results, edited and revised the manuscript, and approved the final version. All measurements were performed at Chongqing Institute of Sports Technology, Chongqing, China.

## Conflict of Interest

The authors declare that the research was conducted in the absence of any commercial or financial relationships that could be construed as a potential conflict of interest.

## Footnotes

1 https://www.biosino.org/node/
